# Network motifs: structure does not determine function

**DOI:** 10.1186/1471-2164-7-108

**Published:** 2006-05-05

**Authors:** Piers J Ingram, Michael PH Stumpf, Jaroslav Stark

**Affiliations:** 1 Department of Mathematics, Imperial College London, 180 Queen's Gate, London, SW7 2AZ, UK; 2 Centre for Bioinformatics, Division of Molecular Biosciences, Wolfson Building, Imperial College London, South Kensington Campus, London, SW7 2AY, UK

## Abstract

**Background:**

A number of publications have recently examined the occurrence and properties of the feed-forward motif in a variety of networks, including those that are of interest in genome biology, such as gene networks. The present work looks in some detail at the dynamics of the bi-fan motif, using systems of ordinary differential equations to model the populations of transcription factors, mRNA and protein, with the aim of extending our understanding of what appear to be important building blocks of gene network structure.

**Results:**

We develop an ordinary differential equation model of the bi-fan motif and analyse variants of the motif corresponding to its behaviour under various conditions. In particular, we examine the effects of different steady and pulsed inputs to five variants of the bifan motif, based on evidence in the literature of bifan motifs found in Saccharomyces cerevisiae (commonly known as baker's yeast). Using this model, we characterize the dynamical behaviour of the bi-fan motif for a wide range of biologically plausible parameters and configurations. We find that there is no *characteristic *behaviour for the motif, and with the correct choice of parameters and of internal structure, very different, indeed even opposite behaviours may be obtained.

**Conclusion:**

Even with this relatively simple model, the bi-fan motif can exhibit a wide range of dynamical responses. This suggests that it is difficult to gain significant insights into biological function simply by considering the connection architecture of a gene network, or its decomposition into simple structural motifs. It is necessary to supplement such structural information by kinetic parameters, or dynamic time series experimental data, both of which are currently difficult to obtain.

## Background

The concept of a *network motif*, introduced by Alon and co-workers [1], has rapidly become one of the central topics of interest in the analysis of complex networks. These networks promise to provide a framework for the understanding of biological processes involving many components such as intra- or inter-cellular networks of interacting genes or proteins. The analysis of such frameworks is one of the key techniques in the rapidly emerging field of systems biology, which makes extensive use of protein interaction, metabolic and gene regulatory networks. Several authors have argued that knowing the structure of these networks, that is knowing the pattern of interactions, will allow us to understand how combinations of genes or proteins interact to achieve specific functional outcomes, and to predict new functional relationships.

A network motif in the sense introduced by Alon and co-workers is a pattern or small sub-graph that occurs more often (at some statistically significant level) in the true network than in an ensemble of networks generated by randomly rewiring the edges in the true network, where the number of nodes and the degree of each node is kept fixed. Of interest are the differences in the frequencies with which network motifs occur in real (biological as well as technological) networks. The recurrent presence of certain motifs has been linked to systematic differences [2] in the functional properties required from networks. In analogy to electric circuits which are built up of smaller modules, such as logic gates, it has been suggested that the motifs in biological networks reflect functional or computational units which combine to regulate the cellular behaviour as a whole. Recently the work of Prill *et al*. [3] has looked at how one aspect of motifs, their stability, influences biological network organisation and specifically the abundance of different motifs in the network.

The detection and enumeration of network motifs has now been followed up by studying the dynamics of corresponding mathematical models of these motifs, especially in the context of transcription regulation networks. These networks aim to describe the links between those genes which code for transcription factors and the genes whose products they control. At the moment, due to the diversity of stimuli a cell/organism can experience, our understanding of the complete sets of regulatory relationships is only preliminary and because of the apparent importance of post-transcriptional regulation, captures only one aspect of the regulatory machinery. Additionally, it must be recalled that these motifs do not exist in isolation within the network, and their behaviour will be heavily influenced by both global and local changes in the cellular environment and the state of the network as a whole. These considerations alone may make attempts to draw positive conclusions about how a motif will behave overly optimistic.

### Network motifs and transcriptional regulation

**Figure 1:**
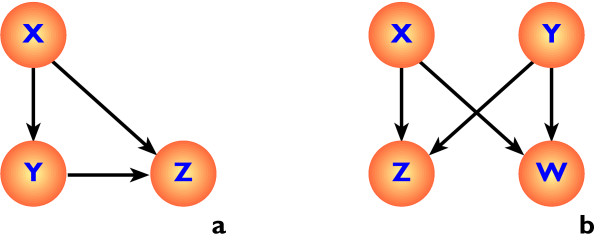
Common motifs in transcription regulation networks, (a) the feed-forward motif, in which the product of gene X regulates both genes Y and Z, whilst Y also regulates gene Z, (b) the bi-fan motif, in which the products of genes X and Y regulate both genes Z and W.

To a great extent, the control of gene transcription is performed by regulatory proteins known as transcription factors, which bind to specific sites on the DNA. Transcription factors may regulate a gene in isolation, but more commonly there are multiple transcription factors acting in concert. The transcription factors are of course themselves products of other (or possibly the same) genes, resulting in a network of interacting regulatory genes. Milo [1] and others have recently looked at ways in which such networks can be broken down into smaller functional units in order to more easily identify structures within the network. It is hoped that the appearance of such smaller units may be indicative of modular structure and efficient design. One of the most important motifs that has hitherto been identified is the feed-forward motif (see Figure 1(a)). A number of recent papers have examined the dynamics of mathematical models of the feed-forward motif [4-6]. Recently, however, it was noted that while the feed-forward loop motif is unusually common, other motifs may be even more prevalent [7]. In particular it was emphasized that when determining the relative statistical significance of the abundance of various motifs, it was important to use an appropriate null "random" model [8]. It was suggested that previously the background structure, that is, physical distance and compartmentalization, had not been adequately taken into account when generating random networks. By using a more sophisticated null model which took into account spatial separation when considering whether nodes would be connected it was found that the "bi-fan" motif was the most prevalent in the transcription regulation networks of both *E. coli *and *S. cerevisiae*. Thus far, however, there has been no detailed study of the dynamics of this motif.

### Bi-fan motifs in S. cerevisiae

**Figure 2:**
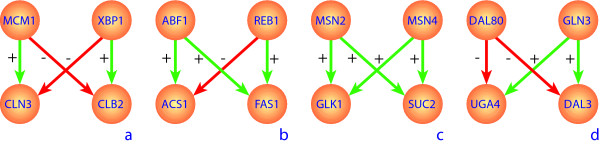
Examplars of bi-fan behaviour observed in the TRANSFAC database for *S. cerevisiae*. Inhibitory interactions are shown in red, while promoting interactions are shown in green.

In Table 1 we list bi-fan motifs extracted from the TRANSFAC database, and in Figure 2 highlight the regulatory relationships reported in the literature for some of these motifs. As is apparent from Table 1, several genes are involved in more than one bi-fan motif. Also, the regulatory interactions documented in the database have been ascertained in a non-uniform way, this simply reflects the non-exhaustive nature of present molecular interaction data-sets.

In Figure 2 repressive interactions are shown in red, while promoting interactions are depicted in green. Even in the small subset of genes for which interaction data was available we were able to find exemplars for a range of distinct bi-fan architectures. We consider and contrast the dynamics of each of these variants.

## Dynamical models of motifs

Given a particular transcription regulation motif, such as those in Figure 1, and straightforward assumptions about the binding kinetics of its constituent molecular species (genes and proteins), we can derive a mathematical model for its dynamics. This then allows analysis of the characteristic responses of these constituents of a motif following an external stimulus. Both deterministic and stochastic models are possible. A number of recent papers have constructed and analyzed models for the feed-forward motif. In particular Mangan and Alon [4] have shown that this single simple motif can exhibit a vast range of different dynamical behaviours.

We believe that these attempts to link structure (of motifs) to function in terms of mathematical models raise a number of interesting questions and problems. The present work looks in some detail at the dynamics of the motifs identified in Table 1. Our analysis follows the approach of [5] for the feed-forward motif and in particular we model the bi-fan motif using a system of ordinary differential equations (ODEs).

We use the number of molecules of the two second tier proteins as a measure of motif behaviour. We look in turn at the four variants of the motif identified in yeast under a series of example dynamical scenarios which reflect the diversity of behaviour that can be demonstrated by this simple motif, starting with coherent motifs, that is, motifs in which every transcription factor acts to promote transcription.

## Results

The motifs in Figure 2 can broadly be separated into two categories – A, B and D are generally referred to as "incoherent motifs", while C is a coherent motif. This nomenclature is due to the arrangement in C in which both inputs act as promoters, in comparison to A and D which are both fully incoherent (both second tier proteins have both promoter and repressor inputs), whilst B may be considered to be partially incoherent, as one of the second tier proteins has incoherent inputs, whilst the other has coherent inputs (the model used in this case is given in equation (1), in which we consider co-operative binding in the case that the order of binding is unimportant). In addition we also add a derivative of the C model which we denote as C', in which both inputs still act as promoters, however the assumption about the way the promoters act is different – we no longer require both promoters to bind for *P*_*W *_to be expressed (in practise it can be seen that these operate as an AND and an OR gate respectively). Further discussion of the many ways in which the transcription factors operates can be modelled can be found in the methods section.

### Constant inputs

We first consider the effect of providing the motif with steady inputs, a biological scenario which corresponds to continued exposure to a either an environmental condition triggering independent factors, or to a constant signal which is split into two signals by the network structure. To examine the various responses, we consider the effect of both signals being turned on continuously at a high level (which we denote in the in Table 3 as a green square), the result of one of the factors occurring at a high level whilst the other is either low (denoted by a yellow square) or off (denoted by red).

In this way one can see the variation in the responses of the two output proteins, which we denote here as *P*_*Z *_and *P*_*W *_for all motifs. In this table, we have simply provided the transcription factors at a reasonably high concentration and quantified the response of each protein after the system has stabilized. All of the simulations are performed with the same kinetic parameters, as detailed in the methods section, and the qualification of a high or low response is based on comparison with the response of other motifs and the response of the other protein. Again, these results are colour-coded for easy comparison, with green, yellow and red again corresponding to high, low or no expression.

We can see that there is significant variation in the characterization of the response of the variants of the motif.

### Response to simultaneous pulses

We next look at the responses of the motif in the case in which both transcription factors are turned on simultaneously for 3600 seconds (an hour), and then turned off again. Again, the precise amplitudes and durations of the protein responses varies greatly, and are dependant on the precise values of the kinetic parameters. Two illustrative cases can be seen in Figure 5, again corresponding to variants A and B of the motifs however. The qualitative results are summarized in Figure 4.

### Staggered pulses

Finally we look at the effect of offset pulses in the levels of transcription factors. In the first case, providing the first transcription factor (denoted by *I*_*X*_) for 3600 seconds, then turning that off, and turning the second factor (*I*_*Y*_) on for 3600 seconds. This is then reversed. The results can be see in Figure 6, again using the colour coding scheme described above.

Two illustrative cases can be seen in Figure 7, corresponding to variants A and B, in the case in which first *I*_*X *_and then *I*_*Y *_are activated.

## Discussion

**Figure 3:**
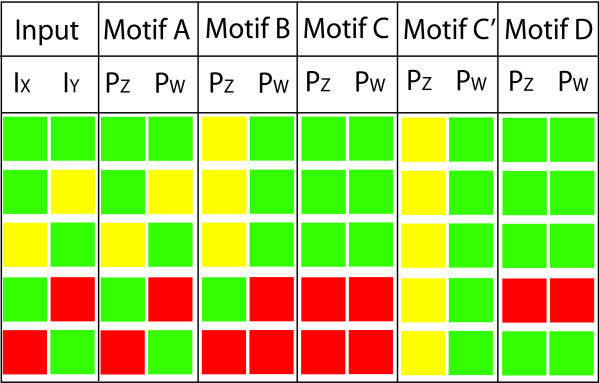
This table shows the variation in the outputs of the four different motifs identified in the literature for a variety of steady inputs. The details of the model used can be found in the methods section, and the values of the parameters used are in Table 2. In the input column, the motif is first exposed to two high inputs, *I*_*X *_and *I*_*Y *_take the value of 100. This is indicated as two green squares. The outputs of the two second tier proteins are then measured after the system has attained equilibrium (after 10,000 seconds), and the outputs are characterized as High, Low or Off, denoted by Green, Yellow or Red squares respectively. On the second row the inputs are changed to be *I*_*X *_high and *I*_*Y *_low (values used were 100 and 10 respectively). In the final two rows the situation is considered in which one of the inputs is turned off (denoted by a red square). What is of most interest is the variation in the pattern of outputs along each row, indicating that the characteristic output of the motif varies qualitatively according to the detailed structure, in addition to the quantitative variation in the outputs.

**Figure 4:**
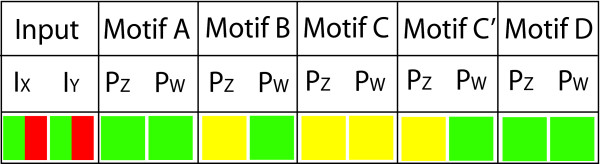
This table shows the variation in the outputs of the different motifs when both inputs are turned on to a high level for 3600 seconds and then abruptly turned off. This is denoted in the inputs as green then red. The outputs are then categorised and colour coded as either off, low or high, corresponding to red, yellow or green. This scenario corresponds to relatively short term exposure to a transcription factor up the transcription network. Again, the details of the model can be found in the methods section, and the parameters used are given in Table 2

**Figure 5:**
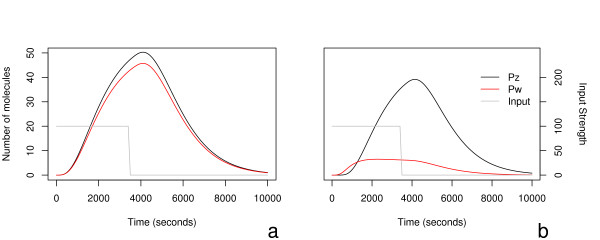
In these two plots we again see the dynamical response of motifs A and B when the motif is excited by simultaneous step functions for 3600 seconds. The two plots share their y-axes, with the scale for the response on the leftmost axis, and the strength of the input signal on the far right axis. Note not only the difference between the two in peak expression, but also the marked difference in total expression, time of peak expression, and behaviour after the peak between the two motifs. The curves in (a) have been separated to enhance readability. To produce the simultaneous steps, both *I*_*X *_and *I*_*Y *_are initially at 0 at *t *= 0, are then set to 100 for 3600 seconds, then again returned to 0. The kinetic parameters used are given in Table 2.

**Figure 6:**
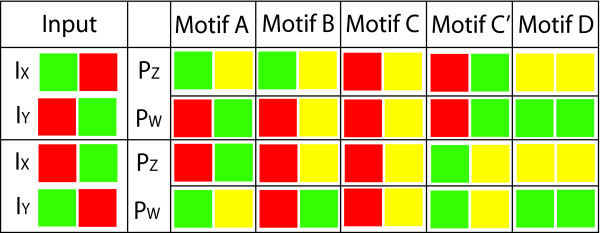
In this table we can see the responses of the different motifs to successive inputs. The first two rows show the effect of first perturbing *I*_*X *_and then *I*_*Y *_with a step function. In the third and fourth rows this is reversed. Again the main feature to notice in this table is the variety in the responses of the motifs modelled. For the purposes of this diagram there were assumed to be two phases of behaviour corresponding to "after" the first perturbation and "after" the second perturbation. The strength of the response is again indicated by Red, Yellow and Green, corresponding to Off, Low or High response. As in the previous cases, the details of the model can be found in the methods section, and the parameters used are given in Table 2.

**Figure 7:**
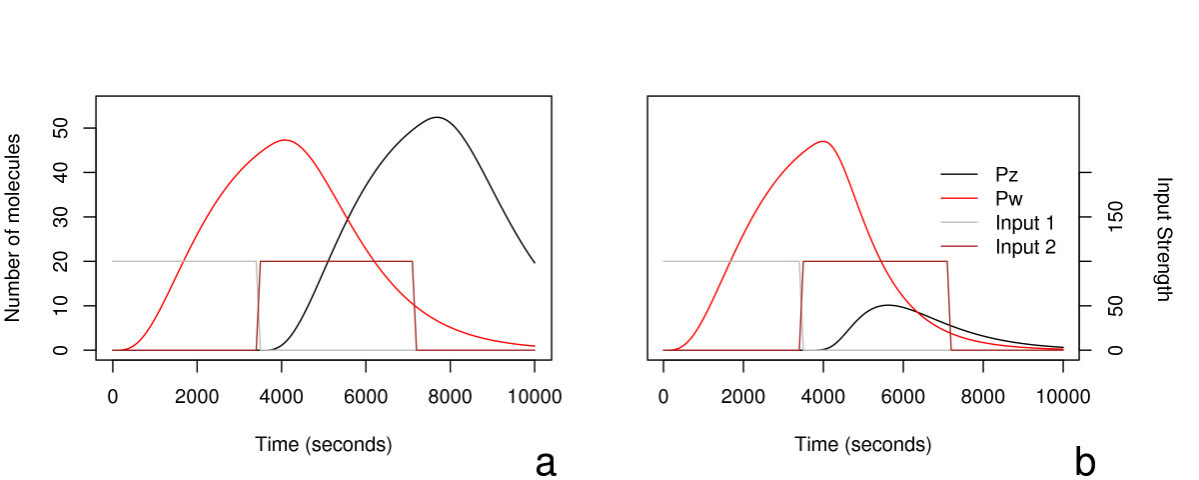
Here we see again the details of the dynamical response of motifs A and B to the inputs. Again, the two plots share y-axes, with the response scale on the left, and input signal strength of the far right axis. In this case, two sucessive step functions of 3600 seconds were used to model activation by first one transcription factor and then the other immediately after. In these figures, *I*_*X *_is increased from 0 to 100 for 3600 seconds, and then at *t *= 3600, *I*_*Y *_is set to 100 for 3600 seconds and *I*_*X *_is turned off. Again the kinetic parameters used in the model can be found in Table 2. Note the significant differences not only in the absolute values of peak expression, but in the shape of the curves.

We have seen that the the bi-fan motif exhibits a rich variety of dynamical behaviour and has the ability to perform a number of potentially useful functions, considering for example Figure 3, the results of steady inputs, we can see that the motif can pass through the inputs as outputs (variant A), act as a logical AND gate (variant C) or an OR gate (variant C'). From this table of responses to the simplest dynamical stimulation we can see already that it is very hard to draw any firm conculsions about the role of a bifan motif without knowing a great deal about its internal structure. Furthermore the simple distinction of coherent or incoherent is also insufficient, as we can see from the opposing behaviours of C and C'. Furthermore, there is great variation in the detailed dynamical response as we have seen from Figures 5 and 7, with differences in total expression levels, steepness of response and timing of peak expression. It must be noted that the majority of the variation in behaviour is not unexpected, and arises as a consequence of the parameters used, however this only exacerbates the difficulties of trying to think of, and use motifs as higher level "functional modules".

## Conclusion

Analysis of many networks in a large number of scenarios, from biological and social networks to technological networks has revealed the presence of motifs, simple patterns which occur with a greater than expected frequency. In the context of biological networks, and specifically transcription regulatory networks, it has been argued [9] that motifs have evolved independently, indicating optimal design. It has been suggested that such motifs may represent "computational elements", and in the case of the feedforward motif, the possibility of the motif acting as a Boolean AND or OR gate has been investigated [4].

Here we have systematically studied a range of dynamical behaviours possible for bi-fan motifs. It had previously been demonstrated that even for the simpler feed-forward motif there is already a vast range of possible dynamical behaviours. For the bi-fan, which is only slightly more complex, we found again that there is a large range of possible response behaviours. Most notably we observed that entirely opposing behaviours (for example, in the case of the responses of variants C and C') can be elicited depending on the nature and strengths of individual interactions within the motif. We were able to identify a variety of combinations of such interactions in the bi-fan motifs found in the transcriptional network of *S. cerevisiae*. In particular, we found examples of both coherent and incoherent architectures. This suggests that simply identifying the presence of particular motifs, without a detailed experimental evaluation of their respective dynamics, is unlikely to offer much insight into the functional properties of real transcriptional networks. In essence this means that knowing the structure of a network, or an inventory of the discrete modules making up that structure, doesn't provide enough information to predict how functional processes occur or how biochemical reactions proceed in a biological system.

Admittedly, our analysis does not take into account the full complexity of real biological bi-fan motifs. This, however, makes the interpretation of bi-fan motif occurrences in nature even more difficult: if simple mathematical models can already demonstrate such different types of behaviour then it is likely that real bi-fan motifs exhibit an even richer repertoire of behaviour. One should also remember that motifs are themselves generally only artificially identified local structures, there is no good reason to believe that their dynamics can necessarily lead to a modularization in understanding the behaviour of transcription networks. Many of the databases used to mine for motifs are based on yeast-2-hybrid experimental data which gives no indication of whether elements of identified structures are active at the same time.

Furthermore we usually lack information about the behaviour of the input signals, essential to understand the relationship of the motif to the network as a whole. Thus, we can conclude that simply knowing the connection structure of this motif is insufficient to give much insight into its function or even its dynamical response. In order to do this, we would need much more detailed experimental information about binding and unbinding rates and other kinetic information. Currently, such experimental data is difficult to determine in a wholesale fashion, making the large scale analysis of the function of transcription networks very problematic.

## Methods

Following [5] we only consider deterministic models and hence use systems of ordinary differential equations (ODEs) to describe the time evolution of the number of molecules of the various constituent species of a bi-fan motif. In many cases the copy numbers of some of these (*e.g*. transcription factors) will be too low for ODEs to capture the dynamics of the real system. In such cases we can use a stochastic model which can be simulated using for instance the Gillespie algorithm [10]. However [5] found that for feed-forward motif the average behaviour of an ensemble of such simulations is well characterised by ODEs describing the mean behaviour. We confirmed this for the bi-fan motif (data not shown). Following the custom in the literature for feed-forward motifs, we refer to the bi-fan network in which all interactions act directly to promote expression as "coherent", and use the term "incoherent" otherwise. We analyse the dynamical response of the motif by observing the expression levels of the proteins in the motif. We assume that the motif is initially at equilibrium with zero input so that in the absence of basal transcription none of the proteins in the motif are initially expressed. We then stimulate the system at one or both inputs for varying periods and at varying strengths, corresponding to different dynamical situations. Our models of transcription regulation were translated into systems of approximately 30 ordinary differential equations [see Additional File 1], which were subsequently solved using Mathematica (Wolfram Research, Urbana Champagne Illinois).

### The bi-fan model

The bi-fan motif, see Figure 1, consists of four regulatory systems, denoted as *X*, *Y*, *Z *and *W*. It is necessary to represent each of the systems in a biologically relevant way and with realistic parameter choices. The model used here follows that of [5], in that each of the systems is composed of a transcriptional part whereby one or more transcription factors bind to promoter regions and regulate the production of mRNA, as well as a translational part whereby the mRNA is translated into protein, which may act as a transcription factor for another regulatory system.

The model is certainly not the simplest available – we could instead have modelled the motif as a Boolean network or with weighted funtions between nodes, however we believe that to do so does not take advantage of the knowledge that has been gained in study of the physical mechanism of gene regulation, and futhermore the model we do use has the advantage that there is considerable experimental data available to justify the choice of rate constants, and not to have used this would have been to not take full advantage of the experimental data. Equally, this model does not attempt to model the many hundreds of intermediate steps involved in each process such as transcription, as the steps introduced would necessarily have been arbitrary, and without experimental data to justify rate constants.

The model attempts to describe the process of gene regulation from transcription binding to protein production in a physically reasonable way. Each system, for example *X*, is represented as having a section of DNA *D*_*X *_which codes for the mRNA *M*_*X*_. First, the transcription factors, which may either be proteins produced by one of the systems in the motif, or by an external signal, bind to the promoter region to form a complex *Q*_*X*_. RNA polymerase molecules *R*_*X *_then bind to this complex as they read the DNA, forming a secondcomplex QX*
 MathType@MTEF@5@5@+=feaafiart1ev1aaatCvAUfKttLearuWrP9MDH5MBPbIqV92AaeXatLxBI9gBaebbnrfifHhDYfgasaacH8akY=wiFfYdH8Gipec8Eeeu0xXdbba9frFj0=OqFfea0dXdd9vqai=hGuQ8kuc9pgc9s8qqaq=dirpe0xb9q8qiLsFr0=vr0=vr0dc8meaabaqaciaacaGaaeqabaqabeGadaaakeaacqWGrbqudaqhaaWcbaGaemiwaGfabaGaeiOkaOcaaaaa@3019@. This complex then breaks down as the reading process completes, releasing in the process *Q*_*X*_, *R*_*X *_and the newly formed mRNA *M*_*X*_. The mRNA molecules are then translated, and copies of the protein *P*_*X *_are produced. The initial numbers of molecules used in the model were: one copy of the DNA for each system and 30 copies of RNA polymerase, all others were set to zero. These reactions are modelled using the law of mass action.

### Modelling the interactions

The detailed modelling of the interactions between the two tiers of proteins can be carried out in a number of ways, all of which will have an effect on the resultant dynamics.

#### Co-operative binding – order unimportant

In this case, considered in the paper [5], both transcription factors act to promote transcription, however they are both needed for transcription to take place. This is modelled by introducing an intermediate species, *T*. The equations for this model are then as follows:

DW+PY⇌k−7k7QWDW+PX⇌k−7k7TWTW+RW⇌k−2k2QW*QW*→k8TW+MW+RW     (1)
 MathType@MTEF@5@5@+=feaafiart1ev1aaatCvAUfKttLearuWrP9MDH5MBPbIqV92AaeXatLxBI9gBaebbnrfifHhDYfgasaacH8akY=wiFfYdH8Gipec8Eeeu0xXdbba9frFj0=OqFfea0dXdd9vqai=hGuQ8kuc9pgc9s8qqaq=dirpe0xb9q8qiLsFr0=vr0=vr0dc8meaabaqaciaacaGaaeqabaqabeGadaaakeaafaqaaeabdaaaaeaacqWGebardaWgaaWcbaGaem4vaCfabeaakiabgUcaRiabdcfaqnaaBaaaleaacqWGzbqwaeqaaaGcbaWaa4GdaSqaaiabdUgaRnaaBaaameaacqaI3aWnaeqaaaWcbaGaem4AaS2aaSbaaWqaaiabgkHiTiabiEda3aqabaaakiaawcCicaGL9gcaaeaacqWGrbqudaWgaaWcbaGaem4vaCfabeaaaOqaaiabdseaenaaBaaaleaacqWGxbWvaeqaaOGaey4kaSIaemiuaa1aaSbaaSqaaiabdIfaybqabaaakeaadaGdoaWcbaGaem4AaS2aaSbaaWqaaiabiEda3aqabaaaleaacqWGRbWAdaWgaaadbaGaeyOeI0IaeG4naCdabeaaaOGaayjWHiaaw2BiaaqaaiabdsfaunaaBaaaleaacqWGxbWvaeqaaaGcbaGaemivaq1aaSbaaSqaaiabdEfaxbqabaGccqGHRaWkcqWGsbGudaWgaaWcbaGaem4vaCfabeaaaOqaamaao4aaleaacqWGRbWAdaWgaaadbaGaeGOmaidabeaaaSqaaiabdUgaRnaaBaaameaacqGHsislcqaIYaGmaeqaaaGccaGLahIaayzVHaaabaGaemyuae1aa0baaSqaaiabdEfaxbqaaiabcQcaQaaaaOqaceqaGnGaaCzcaiabdgfarnaaDaaaleaacqWGxbWvaeaacqGGQaGkaaaakeaadaGdKaWcbaGaem4AaS2aaSbaaWqaaiabiIda4aqabaaaleqakiaawkziaaqaaiabdsfaunaaBaaaleaacqWGxbWvaeqaaOGaey4kaSIaemyta00aaSbaaSqaaiabdEfaxbqabaGccqGHRaWkcqWGsbGudaWgaaWcbaGaem4vaCfabeaaaaGccaWLjaGaaCzcamaabmaabaGaeGymaedacaGLOaGaayzkaaaaaa@78E4@

#### Co-operative binding – order important

DW+PX⇌k−7k7QWQW+PY⇌k−7k7Q′WQ′W+RW⇌k−2k2QW*QW*→k8TW+MW+RW     (2)
 MathType@MTEF@5@5@+=feaafiart1ev1aaatCvAUfKttLearuWrP9MDH5MBPbIqV92AaeXatLxBI9gBaebbnrfifHhDYfgasaacH8akY=wiFfYdH8Gipec8Eeeu0xXdbba9frFj0=OqFfea0dXdd9vqai=hGuQ8kuc9pgc9s8qqaq=dirpe0xb9q8qiLsFr0=vr0=vr0dc8meaabaqaciaacaGaaeqabaqabeGadaaakeGabaaWduaabaqaemaaaaqaaiabdseaenaaBaaaleaacqWGxbWvaeqaaOGaey4kaSIaemiuaa1aaSbaaSqaaiabdIfaybqabaaakeaadaGdoaWcbaGaem4AaS2aaSbaaWqaaiabiEda3aqabaaaleaacqWGRbWAdaWgaaadbaGaeyOeI0IaeG4naCdabeaaaOGaayjWHiaaw2BiaaqaaiabdgfarnaaBaaaleaacqWGxbWvaeqaaaGcbaGaemyuae1aaSbaaSqaaiabdEfaxbqabaGccqGHRaWkcqWGqbaudaWgaaWcbaGaemywaKfabeaaaOqaamaao4aaleaacqWGRbWAdaWgaaadbaGaeG4naCdabeaaaSqaaiabdUgaRnaaBaaameaacqGHsislcqaI3aWnaeqaaaGccaGLahIaayzVHaaabaGafmyuaeLbauaadaWgaaWcbaGaem4vaCfabeaaaOqaaiqbdgfarzaafaWaaSbaaSqaaiabdEfaxbqabaGccqGHRaWkcqWGsbGudaWgaaWcbaGaem4vaCfabeaaaOqaamaao4aaleaacqWGRbWAdaWgaaadbaGaeGOmaidabeaaaSqaaiabdUgaRnaaBaaameaacqGHsislcqaIYaGmaeqaaaGccaGLahIaayzVHaaabaGaemyuae1aa0baaSqaaiabdEfaxbqaaiabcQcaQaaaaOqaceqa8pGaaCzcaiabdgfarnaaDaaaleaacqWGxbWvaeaacqGGQaGkaaaakeaadaGdKaWcbaGaem4AaS2aaSbaaWqaaiabiIda4aqabaaaleqakiaawkziaaqaaiabdsfaunaaBaaaleaacqWGxbWvaeqaaOGaey4kaSIaemyta00aaSbaaSqaaiabdEfaxbqabaGccqGHRaWkcqWGsbGudaWgaaWcbaGaem4vaCfabeaaaaGccaWLjaGaaCzcamaabmaabaGaeGOmaidacaGLOaGaayzkaaaaaa@7A6E@

#### Independent promoters – order unimportant

This is a simpler case than the above, and is simply the case in which both transcription factors act to promote transcription independently. The equations are then as follows:

DW+PY⇌k−7k7QWDW+PX⇌k−7k7QWQW+RW⇌k−2k2QW*QW*→k8TW+MW+RW     (3)
 MathType@MTEF@5@5@+=feaafiart1ev1aaatCvAUfKttLearuWrP9MDH5MBPbIqV92AaeXatLxBI9gBaebbnrfifHhDYfgasaacH8akY=wiFfYdH8Gipec8Eeeu0xXdbba9frFj0=OqFfea0dXdd9vqai=hGuQ8kuc9pgc9s8qqaq=dirpe0xb9q8qiLsFr0=vr0=vr0dc8meaabaqaciaacaGaaeqabaqabeGadaaakeGabaaZduaabaqaemaaaaqaaiabdseaenaaBaaaleaacqWGxbWvaeqaaOGaey4kaSIaemiuaa1aaSbaaSqaaiabdMfazbqabaaakeaadaGdoaWcbaGaem4AaS2aaSbaaWqaaiabiEda3aqabaaaleaacqWGRbWAdaWgaaadbaGaeyOeI0IaeG4naCdabeaaaOGaayjWHiaaw2BiaaqaaiabdgfarnaaBaaaleaacqWGxbWvaeqaaaGcbaGaemiraq0aaSbaaSqaaiabdEfaxbqabaGccqGHRaWkcqWGqbaudaWgaaWcbaGaemiwaGfabeaaaOqaamaao4aaleaacqWGRbWAdaWgaaadbaGaeG4naCdabeaaaSqaaiabdUgaRnaaBaaameaacqGHsislcqaI3aWnaeqaaaGccaGLahIaayzVHaaabaGaemyuae1aaSbaaSqaaiabdEfaxbqabaaakeaacqWGrbqudaWgaaWcbaGaem4vaCfabeaakiabgUcaRiabdkfasnaaBaaaleaacqWGxbWvaeqaaaGcbaWaa4GdaSqaaiabdUgaRnaaBaaameaacqaIYaGmaeqaaaWcbaGaem4AaS2aaSbaaWqaaiabgkHiTiabikdaYaqabaaakiaawcCicaGL9gcaaeaacqWGrbqudaqhaaWcbaGaem4vaCfabaGaeiOkaOcaaaGcbiqabq+acaWLjaGaemyuae1aa0baaSqaaiabdEfaxbqaaiabcQcaQaaaaOqaamaaoqcaleaacqWGRbWAdaWgaaadbaGaeGioaGdabeaaaSqabOGaayPKHaaabaGaemivaq1aaSbaaSqaaiabdEfaxbqabaGccqGHRaWkcqWGnbqtdaWgaaWcbaGaem4vaCfabeaakiabgUcaRiabdkfasnaaBaaaleaacqWGxbWvaeqaaaaakiaaxMaacaWLjaWaaeWaaeaacqaIZaWmaiaawIcacaGLPaaaaaa@7A32@

#### Promoter/repressor combination

In this case the repressor sequesters DNA, making it unavailable for the promoter to bind.

DW+PY⇌k−7k7TWDW+PX⇌k−7k7QWQW+RW⇌k−2k2QW*QW*→k8TW+MW+RW     (4)
 MathType@MTEF@5@5@+=feaafiart1ev1aaatCvAUfKttLearuWrP9MDH5MBPbIqV92AaeXatLxBI9gBaebbnrfifHhDYfgasaacH8akY=wiFfYdH8Gipec8Eeeu0xXdbba9frFj0=OqFfea0dXdd9vqai=hGuQ8kuc9pgc9s8qqaq=dirpe0xb9q8qiLsFr0=vr0=vr0dc8meaabaqaciaacaGaaeqabaqabeGadaaakeaafaqaaeabdaaaaeaacqWGebardaWgaaWcbaGaem4vaCfabeaakiabgUcaRiabdcfaqnaaBaaaleaacqWGzbqwaeqaaaGcbaWaa4GdaSqaaiabdUgaRnaaBaaameaacqaI3aWnaeqaaaWcbaGaem4AaS2aaSbaaWqaaiabgkHiTiabiEda3aqabaaakiaawcCicaGL9gcaaeaacqWGubavdaWgaaWcbaGaem4vaCfabeaaaOqaaiabdseaenaaBaaaleaacqWGxbWvaeqaaOGaey4kaSIaemiuaa1aaSbaaSqaaiabdIfaybqabaaakeaadaGdoaWcbaGaem4AaS2aaSbaaWqaaiabiEda3aqabaaaleaacqWGRbWAdaWgaaadbaGaeyOeI0IaeG4naCdabeaaaOGaayjWHiaaw2BiaaqaaiabdgfarnaaBaaaleaacqWGxbWvaeqaaaGcbaGaemyuae1aaSbaaSqaaiabdEfaxbqabaGccqGHRaWkcqWGsbGudaWgaaWcbaGaem4vaCfabeaaaOqaamaao4aaleaacqWGRbWAdaWgaaadbaGaeGOmaidabeaaaSqaaiabdUgaRnaaBaaameaacqGHsislcqaIYaGmaeqaaaGccaGLahIaayzVHaaabaGaemyuae1aa0baaSqaaiabdEfaxbqaaiabcQcaQaaaaOqaceqaypGaaCzcaiabdgfarnaaDaaaleaacqWGxbWvaeaacqGGQaGkaaaakeaadaGdKaWcbaGaem4AaS2aaSbaaWqaaiabiIda4aqabaaaleqakiaawkziaaqaaiabdsfaunaaBaaaleaacqWGxbWvaeqaaOGaey4kaSIaemyta00aaSbaaSqaaiabdEfaxbqabaGccqGHRaWkcqWGsbGudaWgaaWcbaGaem4vaCfabeaaaaGccaWLjaGaaCzcamaabmaabaGaeGinaqdacaGLOaGaayzkaaaaaa@795C@

#### Promoter/repressor combination – binding order important

**Figure 8:**
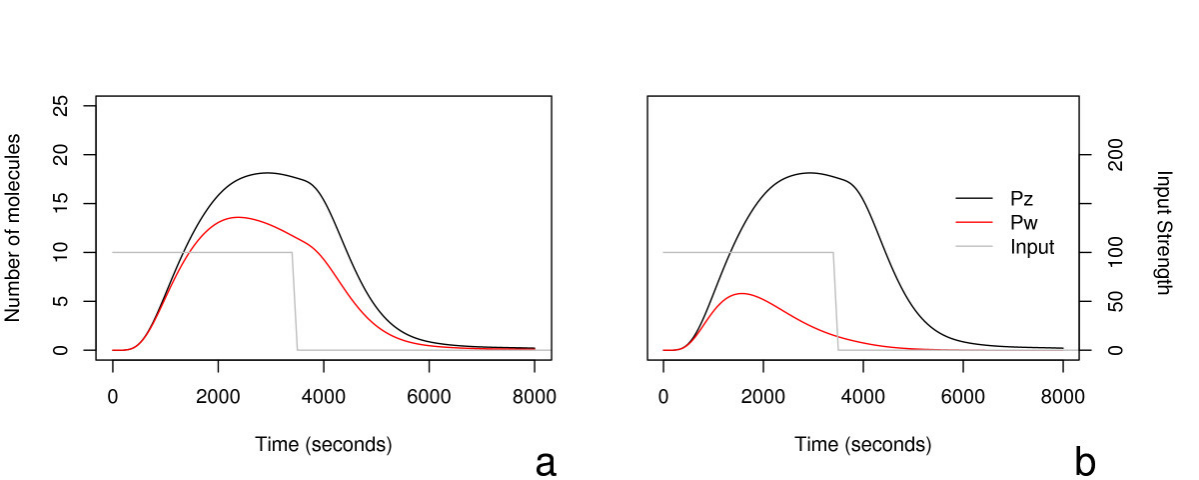
We see the effect of assuming that the production rate of *P*_*W *_is sensitive to the order of binding of *P*_*X *_and *P*_*Y *_to the DNA of gene Z, see text for details. As before, the y-axes are shared. The two expression profiles for *P*_*W *_correspond to inverting the inputs, showing that the motif may able to differentiate between signal combinations. *I*_*X *_and *I*_*Y *_are again modelled as step functions which switch to 0 after 3600 seconds.

In this case, the binding order of *P*_*X *_and *P*_*Y *_to the DNA for gene W now plays a role. If *P*_*Y *_binds first it will block the production of *P*_*W*_, perhaps by altering the conformation of the binding site; if, however, *P*_*X *_binds first then *P*_*Y *_is still required for production of the protein. This has the effect of making *P*_*Y *_a repressor when it binds first to the regulation site. Examples of where this order specific behaviour may occur include the effect of the transcription factor p53 on chromatin structure [11]. Other papers which discuss the importance of binding order include [12] and [13]. We see the effect that this can have on motif behaviour in Figure 8, where the two graphs correspond to high *I*_*X *_and low *I*_*Y *_in (a), and the reverse in (b). Here the motif is apparently able to differentiate between signal combinations.

DW+PY⇌k−7k7QWDW+PX⇌k−7k7TWTW+PY⇌k′−7k′7Q′WQ′W+RW⇌k−2k2QW*QW*→k8Q′W+MW+RW     (5)
 MathType@MTEF@5@5@+=feaafiart1ev1aaatCvAUfKttLearuWrP9MDH5MBPbIqV92AaeXatLxBI9gBaebbnrfifHhDYfgasaacH8akY=wiFfYdH8Gipec8Eeeu0xXdbba9frFj0=OqFfea0dXdd9vqai=hGuQ8kuc9pgc9s8qqaq=dirpe0xb9q8qiLsFr0=vr0=vr0dc8meaabaqaciaacaGaaeqabaqabeGadaaakeaafaqaaeqbdaaaaeaacqWGebardaWgaaWcbaGaem4vaCfabeaakiabgUcaRiabdcfaqnaaBaaaleaacqWGzbqwaeqaaaGcbaWaa4GdaSqaaiabdUgaRnaaBaaameaacqaI3aWnaeqaaaWcbaGaem4AaS2aaSbaaWqaaiabgkHiTiabiEda3aqabaaakiaawcCicaGL9gcaaeaacqWGrbqudaWgaaWcbaGaem4vaCfabeaaaOqaaiabdseaenaaBaaaleaacqWGxbWvaeqaaOGaey4kaSIaemiuaa1aaSbaaSqaaiabdIfaybqabaaakeaadaGdoaWcbaGaem4AaS2aaSbaaWqaaiabiEda3aqabaaaleaacqWGRbWAdaWgaaadbaGaeyOeI0IaeG4naCdabeaaaOGaayjWHiaaw2BiaaqaaiabdsfaunaaBaaaleaacqWGxbWvaeqaaaGcbaGaemivaq1aaSbaaSqaaiabdEfaxbqabaGccqGHRaWkcqWGqbaudaWgaaWcbaGaemywaKfabeaaaOqaamaao4aaleaacuWGRbWAgaqbamaaBaaameaacqaI3aWnaeqaaaWcbaGafm4AaSMbauaadaWgaaadbaGaeyOeI0IaeG4naCdabeaaaOGaayjWHiaaw2BiaaqaaiqbdgfarzaafaWaaSbaaSqaaiabdEfaxbqabaaakeaacuWGrbqugaqbamaaBaaaleaacqWGxbWvaeqaaOGaey4kaSIaemOuai1aaSbaaSqaaiabdEfaxbqabaaakeaadaGdoaWcbaGaem4AaS2aaSbaaWqaaiabikdaYaqabaaaleaacqWGRbWAdaWgaaadbaGaeyOeI0IaeGOmaidabeaaaOGaayjWHiaaw2BiaaqaaiabdgfarnaaDaaaleaacqWGxbWvaeaacqGGQaGkaaaakeGabea2diaaxMaacqWGrbqudaqhaaWcbaGaem4vaCfabaGaeiOkaOcaaaGcbaWaa4ajaSqaaiabdUgaRnaaBaaameaacqaI4aaoaeqaaaWcbeGccaGLsgcaaeaacuWGrbqugaqbamaaBaaaleaacqWGxbWvaeqaaOGaey4kaSIaemyta00aaSbaaSqaaiabdEfaxbqabaGccqGHRaWkcqWGsbGudaWgaaWcbaGaem4vaCfabeaaaaGccaWLjaGaaCzcamaabmaabaGaeGynaudacaGLOaGaayzkaaaaaa@8B77@

#### Illustrative case

With these considerations in mind, we give the full model for variant C, the coherent motif (the other cases are similar). The basic model for *X *and *Y*, where *I*_*X *_and *I*_*Y *_represent the amounts of externally produced transcription factors:

For protein *X*:

DX+IX⇌k−1k1QXQX+RX⇌k−2k2QX*QX*→k3QX+MX+RXMX→k4MX+PXMX→k5∅PX→kX6∅     (6)
 MathType@MTEF@5@5@+=feaafiart1ev1aaatCvAUfKttLearuWrP9MDH5MBPbIqV92AaeXatLxBI9gBaebbnrfifHhDYfgasaacH8akY=wiFfYdH8Gipec8Eeeu0xXdbba9frFj0=OqFfea0dXdd9vqai=hGuQ8kuc9pgc9s8qqaq=dirpe0xb9q8qiLsFr0=vr0=vr0dc8meaabaqaciaacaGaaeqabaqabeGadaaakeaafaqaaeGbdaaaaeaacqWGebardaWgaaWcbaGaemiwaGfabeaakiabgUcaRiabdMeajnaaBaaaleaacqWGybawaeqaaaGcbaWaa4GdaSqaaiabdUgaRnaaBaaameaacqaIXaqmaeqaaaWcbaGaem4AaS2aaSbaaWqaaiabgkHiTiabigdaXaqabaaakiaawcCicaGL9gcaaeaacqWGrbqudaWgaaWcbaGaemiwaGfabeaaaOqaaiabdgfarnaaBaaaleaacqWGybawaeqaaOGaey4kaSIaemOuai1aaSbaaSqaaiabdIfaybqabaaakeaadaGdoaWcbaGaem4AaS2aaSbaaWqaaiabikdaYaqabaaaleaacqWGRbWAdaWgaaadbaGaeyOeI0IaeGOmaidabeaaaOGaayjWHiaaw2BiaaqaaiabdgfarnaaDaaaleaacqWGybawaeaacqGGQaGkaaaakeGabaagdiaaxMaacqWGrbqudaqhaaWcbaGaemiwaGfabaGaeiOkaOcaaaGcbaWaa4ajaSqaaiabdUgaRnaaBaaameaacqaIZaWmaeqaaaWcbeGccaGLsgcaaeaacqWGrbqudaWgaaWcbaGaemiwaGfabeaakiabgUcaRiabd2eannaaBaaaleaacqWGybawaeqaaOGaey4kaSIaemOuai1aaSbaaSqaaiabdIfaybqabaaakeGabaa9ciaaxMaacqWGnbqtdaWgaaWcbaGaemiwaGfabeaaaOqaamaaoqcaleaacqWGRbWAdaWgaaadbaGaeGinaqdabeaaaSqabOGaayPKHaaabaGaemyta00aaSbaaSqaaiabdIfaybqabaGccqGHRaWkcqWGqbaudaWgaaWcbaGaemiwaGfabeaaaOqaceaammGaaCzcaiabd2eannaaBaaaleaacqWGybawaeqaaaGcbaWaa4ajaSqaaiabdUgaRnaaBaaameaacqaI1aqnaeqaaaWcbeGccaGLsgcaaeaacqGHfiIXaeGabaamdiaaxMaacqWGqbaudaWgaaWcbaGaemiwaGfabeaaaOqaamaaoqcaleaacqWGRbWAdaWgaaadbaGaemiwaGLaeGOnaydabeaaaSqabOGaayPKHaaabaGaeyybIymaaiaaxMaacaWLjaWaaeWaaeaacqaI2aGnaiaawIcacaGLPaaaaaa@884D@

For protein *Y*:

DY+IY⇌k−1k1QYQY+RY⇌k−2k2QY*QY*→k3QY+MY+RYMY→k4MY+PYMY→k5∅PY→kY6∅     (7)
 MathType@MTEF@5@5@+=feaafiart1ev1aaatCvAUfKttLearuWrP9MDH5MBPbIqV92AaeXatLxBI9gBaebbnrfifHhDYfgasaacH8akY=wiFfYdH8Gipec8Eeeu0xXdbba9frFj0=OqFfea0dXdd9vqai=hGuQ8kuc9pgc9s8qqaq=dirpe0xb9q8qiLsFr0=vr0=vr0dc8meaabaqaciaacaGaaeqabaqabeGadaaakeaafaqaaeGbdaaaaeaacqWGebardaWgaaWcbaGaemywaKfabeaakiabgUcaRiabdMeajnaaBaaaleaacqWGzbqwaeqaaaGcbaWaa4GdaSqaaiabdUgaRnaaBaaameaacqaIXaqmaeqaaaWcbaGaem4AaS2aaSbaaWqaaiabgkHiTiabigdaXaqabaaakiaawcCicaGL9gcaaeaacqWGrbqudaWgaaWcbaGaemywaKfabeaaaOqaaiabdgfarnaaBaaaleaacqWGzbqwaeqaaOGaey4kaSIaemOuai1aaSbaaSqaaiabdMfazbqabaaakeaadaGdoaWcbaGaem4AaS2aaSbaaWqaaiabikdaYaqabaaaleaacqWGRbWAdaWgaaadbaGaeyOeI0IaeGOmaidabeaaaOGaayjWHiaaw2BiaaqaaiabdgfarnaaDaaaleaacqWGzbqwaeaacqGGQaGkaaaakeGabaagdiaaxMaacqWGrbqudaqhaaWcbaGaemywaKfabaGaeiOkaOcaaaGcbaWaa4ajaSqaaiabdUgaRnaaBaaameaacqaIZaWmaeqaaaWcbeGccaGLsgcaaeaacqWGrbqudaWgaaWcbaGaemywaKfabeaakiabgUcaRiabd2eannaaBaaaleaacqWGzbqwaeqaaOGaey4kaSIaemOuai1aaSbaaSqaaiabdMfazbqabaaakeGabaa9ciaaxMaacqWGnbqtdaWgaaWcbaGaemywaKfabeaaaOqaamaaoqcaleaacqWGRbWAdaWgaaadbaGaeGinaqdabeaaaSqabOGaayPKHaaabaGaemyta00aaSbaaSqaaiabdMfazbqabaGccqGHRaWkcqWGqbaudaWgaaWcbaGaemywaKfabeaaaOqaceaammGaaCzcaiabd2eannaaBaaaleaacqWGzbqwaeqaaaGcbaWaa4ajaSqaaiabdUgaRnaaBaaameaacqaI1aqnaeqaaaWcbeGccaGLsgcaaeaacqGHfiIXaeGabaamdiaaxMaacqWGqbaudaWgaaWcbaGaemywaKfabeaaaOqaamaaoqcaleaacqWGRbWAdaWgaaadbaGaemywaKLaeGOnaydabeaaaSqabOGaayPKHaaabaGaeyybIymaaiaaxMaacaWLjaWaaeWaaeaacqaI3aWnaiaawIcacaGLPaaaaaa@886F@

In the case in which both transcription factors act as promoters, and in which binding order is unimportant, that is, Equation (1), then the equations for proteins *Z *and *W *then become:

DZ+PX⇌k−7k7QZQZ+PY⇌k′−7k′7Q′ZDZ+PY⇌k−7k7TZTZ+PX⇌k′−7k′7Q′ZQ′Z+RZ⇌k−2k2QZ*QZ*→k8Q′Z+MZ+RZMZ→k4MZ+PZMZ→k5∅PZ→kZ6∅     (8)
 MathType@MTEF@5@5@+=feaafiart1ev1aaatCvAUfKttLearuWrP9MDH5MBPbIqV92AaeXatLxBI9gBaebbnrfifHhDYfgasaacH8akY=wiFfYdH8Gipec8Eeeu0xXdbba9frFj0=OqFfea0dXdd9vqai=hGuQ8kuc9pgc9s8qqaq=dirpe0xb9q8qiLsFr0=vr0=vr0dc8meaabaqaciaacaGaaeqabaqabeGadaaakeaafaqaaeqcdaaaaaqaaiabdseaenaaBaaaleaacqWGAbGwaeqaaOGaey4kaSIaemiuaa1aaSbaaSqaaiabdIfaybqabaaakeaadaGdoaWcbaGaem4AaS2aaSbaaWqaaiabiEda3aqabaaaleaacqWGRbWAdaWgaaadbaGaeyOeI0IaeG4naCdabeaaaOGaayjWHiaaw2BiaaqaaiabdgfarnaaBaaaleaacqWGAbGwaeqaaaGcbaGaemyuae1aaSbaaSqaaiabdQfaAbqabaGccqGHRaWkcqWGqbaudaWgaaWcbaGaemywaKfabeaaaOqaamaao4aaleaacuWGRbWAgaqbamaaBaaameaacqaI3aWnaeqaaaWcbaGafm4AaSMbauaadaWgaaadbaGaeyOeI0IaeG4naCdabeaaaOGaayjWHiaaw2BiaaqaaiqbdgfarzaafaWaaSbaaSqaaiabdQfaAbqabaaakeaacqWGebardaWgaaWcbaGaemOwaOfabeaakiabgUcaRiabdcfaqnaaBaaaleaacqWGzbqwaeqaaaGcbaWaa4GdaSqaaiabdUgaRnaaBaaameaacqaI3aWnaeqaaaWcbaGaem4AaS2aaSbaaWqaaiabgkHiTiabiEda3aqabaaakiaawcCicaGL9gcaaeaacqWGubavdaWgaaWcbaGaemOwaOfabeaaaOqaaiabdsfaunaaBaaaleaacqWGAbGwaeqaaOGaey4kaSIaemiuaa1aaSbaaSqaaiabdIfaybqabaaakeaadaGdoaWcbaGafm4AaSMbauaadaWgaaadbaGaeG4naCdabeaaaSqaaiqbdUgaRzaafaWaaSbaaWqaaiabgkHiTiabiEda3aqabaaakiaawcCicaGL9gcaaeaacuWGrbqugaqbamaaBaaaleaacqWGAbGwaeqaaaGcbaGafmyuaeLbauaadaWgaaWcbaGaemOwaOfabeaakiabgUcaRiabdkfasnaaBaaaleaacqWGAbGwaeqaaaGcbaWaa4GdaSqaaiabdUgaRnaaBaaameaacqaIYaGmaeqaaaWcbaGaem4AaS2aaSbaaWqaaiabgkHiTiabikdaYaqabaaakiaawcCicaGL9gcaaeaacqWGrbqudaqhaaWcbaGaemOwaOfabaGaeiOkaOcaaaGcbiqaaWQacaWLjaGaemyuae1aa0baaSqaaiabdQfaAbqaaiabcQcaQaaaaOqaamaaoqcaleaacqWGRbWAdaWgaaadbaGaeGioaGdabeaaaSqabOGaayPKHaaabaGafmyuaeLbauaadaWgaaWcbaGaemOwaOfabeaakiabgUcaRiabd2eannaaBaaaleaacqWGAbGwaeqaaOGaey4kaSIaemOuai1aaSbaaSqaaiabdQfaAbqabaaakeGabaaXciaaxMaacqWGnbqtdaWgaaWcbaGaemOwaOfabeaaaOqaamaaoqcaleaacqWGRbWAdaWgaaadbaGaeGinaqdabeaaaSqabOGaayPKHaaabaGaemyta00aaSbaaSqaaiabdQfaAbqabaGccqGHRaWkcqWGqbaudaWgaaWcbaGaemOwaOfabeaaaOqaceaaelGaaCzcaiabd2eannaaBaaaleaacqWGAbGwaeqaaaGcbaWaa4ajaSqaaiabdUgaRnaaBaaameaacqaI1aqnaeqaaaWcbeGccaGLsgcaaeaacqGHfiIXaeGabaa9ciaaxMaacqWGqbaudaWgaaWcbaGaemOwaOfabeaaaOqaamaaoqcaleaacqWGRbWAdaWgaaadbaGaemOwaOLaeGOnaydabeaaaSqabOGaayPKHaaabaGaeyybIymaaiaaxMaacaWLjaWaaeWaaeaacqaI4aaoaiaawIcacaGLPaaaaaa@C0A5@

DW+PY⇌k−7k7QWQW+PY⇌k′−7k′7Q′WDW+PX⇌k−7k7TWTW+PY⇌k′−7k′7Q′WQ′W+RW⇌k−2k2QW*QW*→k8Q′W+MW+RWMW→k4MW+PWMW→k5∅PW→kW6∅     (9)
 MathType@MTEF@5@5@+=feaafiart1ev1aaatCvAUfKttLearuWrP9MDH5MBPbIqV92AaeXatLxBI9gBaebbnrfifHhDYfgasaacH8akY=wiFfYdH8Gipec8Eeeu0xXdbba9frFj0=OqFfea0dXdd9vqai=hGuQ8kuc9pgc9s8qqaq=dirpe0xb9q8qiLsFr0=vr0=vr0dc8meaabaqaciaacaGaaeqabaqabeGadaaakeaafaqaaeqcdaaaaaqaaiabdseaenaaBaaaleaacqWGxbWvaeqaaOGaey4kaSIaemiuaa1aaSbaaSqaaiabdMfazbqabaaakeaadaGdoaWcbaGaem4AaS2aaSbaaWqaaiabiEda3aqabaaaleaacqWGRbWAdaWgaaadbaGaeyOeI0IaeG4naCdabeaaaOGaayjWHiaaw2BiaaqaaiabdgfarnaaBaaaleaacqWGxbWvaeqaaaGcbaGaemyuae1aaSbaaSqaaiabdEfaxbqabaGccqGHRaWkcqWGqbaudaWgaaWcbaGaemywaKfabeaaaOqaamaao4aaleaacuWGRbWAgaqbamaaBaaameaacqaI3aWnaeqaaaWcbaGafm4AaSMbauaadaWgaaadbaGaeyOeI0IaeG4naCdabeaaaOGaayjWHiaaw2BiaaqaaiqbdgfarzaafaWaaSbaaSqaaiabdEfaxbqabaaakeaacqWGebardaWgaaWcbaGaem4vaCfabeaakiabgUcaRiabdcfaqnaaBaaaleaacqWGybawaeqaaaGcbaWaa4GdaSqaaiabdUgaRnaaBaaameaacqaI3aWnaeqaaaWcbaGaem4AaS2aaSbaaWqaaiabgkHiTiabiEda3aqabaaakiaawcCicaGL9gcaaeaacqWGubavdaWgaaWcbaGaem4vaCfabeaaaOqaaiabdsfaunaaBaaaleaacqWGxbWvaeqaaOGaey4kaSIaemiuaa1aaSbaaSqaaiabdMfazbqabaaakeaadaGdoaWcbaGafm4AaSMbauaadaWgaaadbaGaeG4naCdabeaaaSqaaiqbdUgaRzaafaWaaSbaaWqaaiabgkHiTiabiEda3aqabaaakiaawcCicaGL9gcaaeaacuWGrbqugaqbamaaBaaaleaacqWGxbWvaeqaaaGcbaGafmyuaeLbauaadaWgaaWcbaGaem4vaCfabeaakiabgUcaRiabdkfasnaaBaaaleaacqWGxbWvaeqaaaGcbaWaa4GdaSqaaiabdUgaRnaaBaaameaacqaIYaGmaeqaaaWcbaGaem4AaS2aaSbaaWqaaiabgkHiTiabikdaYaqabaaakiaawcCicaGL9gcaaeaacqWGrbqudaqhaaWcbaGaem4vaCfabaGaeiOkaOcaaaGcbiqaaWQacaWLjaGaemyuae1aa0baaSqaaiabdEfaxbqaaiabcQcaQaaaaOqaamaaoqcaleaacqWGRbWAdaWgaaadbaGaeGioaGdabeaaaSqabOGaayPKHaaabaGafmyuaeLbauaadaWgaaWcbaGaem4vaCfabeaakiabgUcaRiabd2eannaaBaaaleaacqWGxbWvaeqaaOGaey4kaSIaemOuai1aaSbaaSqaaiabdEfaxbqabaaakeGabaaXciaaxMaacqWGnbqtdaWgaaWcbaGaem4vaCfabeaaaOqaamaaoqcaleaacqWGRbWAdaWgaaadbaGaeGinaqdabeaaaSqabOGaayPKHaaabaGaemyta00aaSbaaSqaaiabdEfaxbqabaGccqGHRaWkcqWGqbaudaWgaaWcbaGaem4vaCfabeaaaOqaceaaelGaaCzcaiabd2eannaaBaaaleaacqWGxbWvaeqaaaGcbaWaa4ajaSqaaiabdUgaRnaaBaaameaacqaI1aqnaeqaaaWcbeGccaGLsgcaaeaacqGHfiIXaeGabaa9ciaaxMaacqWGqbaudaWgaaWcbaGaem4vaCfabeaaaOqaamaaoqcaleaacqWGRbWAdaWgaaadbaGaem4vaCLaeGOnaydabeaaaSqabOGaayPKHaaabaGaeyybIymaaiaaxMaacaWLjaWaaeWaaeaacqaI5aqoaiaawIcacaGLPaaaaaa@C02B@

The parameters used for the rates of transcription and translation are based on the derivations in [14], and were obtained from experimentally determined rates [15]. The values used for the basic model are shown in Table 2.

## Authors' contributions

PJI performed the analysis. PJI, MPHS and JS designed the study and jointly wrote the paper.

## Appendix: motifs and networks

A *network *or a *graph *is a mathematical object consisting of a set *V *of vertices, and a set *E *of edges connecting vertices. If the edges have arrows or directions then the network is called a directed network. Many different types of data and relationships may be represented in this way. Examples of undirected networks are networks of pairs of proteins which are known to interact. The networks considered in this paper have as their vertices genes which regulate or are regulated by the product of other genes. The edges then represent the relationship of control, and therefore the network is a directed network, with the direction of the edges indicating the direction of control. Figure A1 is an example of a directed network. Motifs, introduced in [1] are small sub-networks or patterns of vertices and edges which occur with in the network. A motif is considered to be interesting if it occurs unusually frequently in the network. In Figure A1 a (feed-forward) motif is highlighted in colour.

## Appendix figure

A directed network with a feed-forward motif highlighted in colour. In the case of a transcription regulation network the arrows indicate the direction of transcriptional control.

## Supplementary Material

Additional File 1 Supplementary material with details of the differential equations used to model a coherent bi-fan motif in which there is full cooperativity. Click here for file

## Figures and Tables

**Table 1:** Regulators and regulated genes observed in bifan motifs in *S. cerevisiae *and references to the relevant literature.

**Regulator 1**	**Regulator 2**	**Regulated 1**	**Regulated 2**	**References**
MCM1	XBP1	CLN3	CLB2	[16], [17]
ABF1	REB1	ACS1	FAS1	[18], [19], [20]
MSN2	MED8	GLK1	SUC2	[21], [22], [23], [24], [25]
MSN2	MSN4	GLK1	SUC2	[21], [22]
MED8	MSN4	GLK1	SUC2	[21], [24]
DAL80	GLN3	UGA4	DAL3	[26], [27], [28], [29], [30]
SK01	MIG1	ENA1	SUC2	[25], [23], [25], [31]
RAP1	GCR1	ENO1	ENO2	[32], [33], [34], [21], [35], [36], [37]
ABF1	RAP1	ENO2	FAS1	[36], [37], [33]
CAT8	SIP4	FBP1	MDH2	[38], [39], [40], [41]

**Table 2:** Kinetic parameters used in the model. Other parameters not given here are low number mulitples of the corresponding parameter, and were altered as described in the text.

**Parameter**	**Value**	**Interpretation**
*k*_1_, *k*_-1_	0.012, 0.9	Promoter binding/unbinding
*k*_2_, *k*_-2_	0.038, 0.3	RNA polymerase – DNA/promoter binding/unbinding
*k*_3_	0.039	Transcription rate for X & Y
*k*_4_	0.043	Translation rate for X & Y
*k*_5_	0.0039	mRNA decay rate
*k*_6_	0.0007	Protein decay rate
*k*_7_, *k*_-7_	0.012, 0.9	Promoter binding/unbinding for Z & W
*k*_8_	0.039	Transcription rate for Z & W

## References

[B1] Milo R, Shen-Orr S, Itzkovitz S, Kashtan N, Chklovskii D, Alon U (2002). Network motifs: simple building blocks of complex networks. Science.

[B2] Milo R, Itzkovitz S, Kashtan N, Levitt R, Shen-Orr S, Ayzenshtat I, Sheffer M, Alon U (2004). Superfamilies of evolved and designed networks. Science.

[B3] Prill R, Iglesias P, Levchenko A (2005). Dynamic Properties of Network Motifs Contribute to Biological Network Organization. PLoS Biol.

[B4] Mangan S, Alon U (2003). Structure and function of the feed-forward loop network motif. Proc Natl Acad Sci U S A.

[B5] Hayot F, Jayaprakash C (2005). A feedforward loop motif in transcriptional regulation: induction and repression. J Theor Biol.

[B6] Mangan S, Zaslaver A, Alon U (2003). The coherent feedforward loop serves as a sign-sensitive delay element in transcription networks. J Mol Biol.

[B7] Artzy-Randrup Y, Fleishman SJ, Ben-Tal N, Stone L (2004). Comment on "Network motifs: simple building blocks of complex networks" and "Superfamilies of evolved and designed networks". Science.

[B8] Huang S (2004). Back to the biology in systems biology: what can we learn from biomolecular networks?. Brief Fund Genomic Proteomic.

[B9] Conant GC, Wagner A (2003). Convergent evolution of gene circuits. Nat Genet.

[B10] Gillespie D (1977). Exact stochastic simulation of coupled chemical reactions. J Phys Chem.

[B11] Ogden SK, Lee KC, Wernke-Dollries K, Stratton SA, Aronow B, Barton MC (2001). p53 targets chromatin structure alteration to repress alpha-fetoprotein gene expression. J Biol Chem.

[B12] Hiroi H, Christenson LK, Chang L, Sammel MD, Berger SL, Strauss JFr (2004). Temporal and spatial changes in transcription factor binding and histone modifications at the steroidogenic acute regulatory protein (stAR) locus associated with stAR transcription. Mol Endocrinol.

[B13] Cosma MP (2002). Ordered recruitment: gene-specific mechanism of transcription activation. Mol Cell.

[B14] Bundschuh R, Hayot F, Jayaprakash C (2003). The role of dimerization in noise reduction of simple genetic networks. J Theor Biol.

[B15] Ptashne M, Jeffrey A, Johnson AD, Maurer R, Meyer BJ, Pabo CO, Roberts TM, Sauer RT (1980). How the lambda repressor and cro work. Cell.

[B16] Mai B, Breeden L (2000). CLN1 and its repression by Xbp1 are important for efficient sporulation in budding yeast. Mol Cell Biol.

[B17] Althoefer H, Schleiffer A, Wassmann K, Nordheim A, Ammerer G (1995). Mcm1 is required to coordinate G2-specific transcription in Saccharomyces cerevisiae. Mol Cell Biol.

[B18] Kratzer S, Schuller HJ (1997). Transcriptional control of the yeast acetyl-CoA synthetase gene, ACS1, by the positive regulators CAT8 and ADR1 and the pleiotropic repressor UME6. Mol Microbiol.

[B19] Kratzer S, Schuller HJ (1995). Carbon source-dependent regulation of the acety1-coenzyme A synthetase-encoding gene ACS1 from Saccharomyces cerevisiae. Gene.

[B20] Schuller HJ, Schutz A, Knab S, Hoffmann B, Schweizer E (1994). Importance of general regulatory factors Rap1p, Abf1p and Reb1p for the activation of yeast fatty acid synthase genes FAS1 and FAS2. Eur J Biochem.

[B21] Herrero P, Flores L, de la Cera T, Moreno F (1999). Functional characterization of transcriptional regulatory elements in the upstream region of the yeast GLK1 gene. Biochem J.

[B22] Moreno-Herrero F, Herrero P, Colchero J, Baro AM, Moreno F (1999). Analysis by atomic force microscopy of Med8 binding to cis-acting regulatory elements of the SUC2 and HXK2 genes of saccharomyces cerevisiae. FEBS Lett.

[B23] Bu Y, Schmidt MC (1998). Identification of cis-acting elements in the SUC2 promoter of Saccharomyces cerevisiae required for activation of transcription. Nucleic Acids Res.

[B24] Estruch F, Carlson M (1993). Two homologous zinc finger genes identified by multicopy suppression in a SNF1 protein kinase mutant of Saccharomyces cerevisiae. Mol Cell Biol.

[B25] Nehlin JO, Carlberg M, Ronne H (1992). Yeast SKO1 gene encodes a bZIP protein that binds to the CRE motif and acts as a repressor of transcription. Nucleic Acids Res.

[B26] Coffman JA, Rai R, Loprete DM, Cunningham T, Svetlov V, Cooper TG (1997). Cross regulation of four GATA factors that control nitrogen catabolic gene expression in Saccharomyces cerevisiae. J Bacteriol.

[B27] Cunningham TS, Svetlov VV, Rai R, Smart W, Cooper TG (1996). G1n3p is capable of binding to UAS(NTR) elements and activating transcription in Saccharomyces cerevisiae. J Bacteriol.

[B28] Talibi D, Grenson M, Andre B (1995). Cis- and trans-acting elements determining induction of the genes of the gamma-aminobutyrate (GABA) utilization pathway in Saccharomyces cerevisiae. Nucleic Acids Res.

[B29] Andre B, Talibi D, Soussi Boudekou S, Hein C, Vissers S, Coornaert D (1995). Two mutually exclusive regulatory systems inhibit UASGATA, a cluster of 5'-GAT(A/T)A-3' upstream from the UGA4 gene of Saccharomyces cerevisiae. Nucleic Acids Res.

[B30] Cunningham TS, Cooper TG (1993). The Saccharomyces cerevisiae DAL80 repressor protein binds to multiple copies of GATAA-containing sequences (URSGATA). J Bacteriol.

[B31] Proft M, Serrano R (1999). Repressors and upstream repressing sequences of the stress-regulated ENA1 gene in Saccharomyces cerevisiae: bZIP protein Sko1p confers HOG-dependent osmotic regulation. Mol Cell Biol.

[B32] Huie MA, Scott EW, Drazinic CM, Lopez MC, Hornstra IK, Yang TP, Baker HV (1992). Characterization of the DNA-binding activity of GCR1: in vivo evidence for two GCR1-binding sites in the upstream activating sequence of TPI of Saccharomyces cerevisiae. Mol Cell Biol.

[B33] Brindle PK, Holland JP, Willett CE, Innis MA, Holland MJ (1990). Multiple factors bind the upstream activation sites of the yeast enolase genes ENO1 and ENO2: ABFI protein, like repressor activator protein RAP1, binds cis-acting sequences which modulate repression or activation of transcription. Mol Cell Biol.

[B34] Machida M, Jigami Y, Tanaka H (1989). Purification and characterization of a nuclear factor which binds specifically to the upstream activation sequence of Saccharomyces cerevisiae enolase 1 gene. Eur J Biochem.

[B35] Machida M, Uemura H, Jigami Y, Tanaka H (1988). The protein factor which binds to the upstream activating sequence of Saccharomyces cerevisiae ENO1 gene. Nucleic Acids Res.

[B36] Willett CE, Gelfman CM, Holland MJ (1993). A complex regulatory element from the yeast gene ENO2 modulates GCRl-dependent transcriptional activation. Mol Cell Biol.

[B37] Holland JP, Brindle PK, Holland MJ (1990). Sequences within an upstream activation site in the yeast enolase gene ENO2 modulate repression of ENO2 expression in strains carrying a null mutation in the positive regulatory gene GCR1. Mol Cell Biol.

[B38] Zaragoza O, Vincent O, Gancedo JM (2001). Regulatory elements in the FBP1 promoter respond differently to glucose-dependent signals in Saccharomyces cerevisiae. Biochem J.

[B39] Vincent O, Carlson M (1998). Sip4, a Snf1 kinase-dependent transcriptional activator, binds to the carbon source-responsive element of gluconeogenic genes. EMBO J.

[B40] Scholer A, Schuller HJ (1994). A carbon source-responsive promoter element necessary for activation of the isocitrate lyase gene ICL1 is common to genes of the gluconeogenic pathway in the yeast Saccharomyces cerevisiae. Mol Cell Biol.

[B41] Roth S, Schuller HJ (2001). Cat8 and Sip4 mediate regulated transcriptional activation of the yeast malate dehydrogenase gene MDH2 by three carbon source-responsive promoter elements. Yeast.

