# Supplementary material for: Network motifs: structure does not determine function
Source: BMC Genomics. 2006 May 5;7:108. doi: 10.1186/1471-2164-7-108 (PMC1488845; doi:10.1186/1471-2164-7-108)
Supplement: Additional File 1 — Supplementary material with details of the differential equations used to model a coherent bi-fan motif in which there is full cooperativity. [file 1471-2164-7-108-S1.pdf]

Supplementary Material: Ordinary Differential Equations describing model

The following are the system of coupled ordinary differential equations which model the coherent bifan network, for which full cooperativity occurs. In this simple case, the kinetic parameters used for all four genes are identical. Note in particular the coupling terms between the DNA elements, D_Z and D_W and the regulatory proteins P_X and P_Y. The functions In_X and In_Y are modelled as offset Heaviside (step funtions), eg:

$$In_X = 100\Theta(3600 - t)$$

$$\begin{aligned}
\frac{d}{dt}D_X(t) &= -k_1 D_X(t) In_X(t) + k_{-1} Q_X(t) \\
\frac{d}{dt}Q_X(t) &= k_1 D_X(t) In_X(t) - k_{-1} Q_X(t) - k_2 Q_X(t) R_X(t) + k_{-2} Q_X^*(t) + k_3 Q_X^*(t) \\
\frac{d}{dt}Q_X^*(t) &= k_2 Q_X(t) R_X(t) - k_{-2} Q_X^*(t) - k_3 Q_X^*(t) \\
\frac{d}{dt}R_X(t) &= -k_2 Q_X(t) R_X(t) + k_{-2} Q_X^*(t) + k_3 Q_X^*(t) \\
\frac{d}{dt}M_X(t) &= k_3 Q_X^*(t) - k_5 M_X(t) \\
\frac{d}{dt}P_X(t) &= k_4 M_X(t) - k_6 P_X(t) + (-k_1 D_W(t) P_X(t) + k_{-1} T_W(t)) + (-k_1 Q_W(t) P_X(t) + k_{-1} Q'_W(t)) \\
&\quad + (-k_1 D_Z(t) P_X(t) + k_{-1} T_Z(t)) + (-k_1 Q_Z(t) P_X(t) + k_{-1} Q'_Z(t))
\end{aligned} \tag{1}$$

$$\begin{aligned}
\frac{d}{dt}D_Y(t) &= -k_1 D_Y(t) In_Y(t) + k_{-1} Q_Y(t) \\
\frac{d}{dt}Q_Y(t) &= k_1 D_Y(t) In_Y(t) - k_{-1} Q_Y(t) - k_2 Q_Y(t) R_Y(t) + k_{-2} Q_Y^*(t) + k_3 Q_Y^*(t) \\
\frac{d}{dt}Q_Y^*(t) &= k_2 Q_Y(t) R_Y(t) - k_{-2} Q_Y^*(t) - k_3 Q_Y^*(t) \\
\frac{d}{dt}R_Y(t) &= -k_2 Q_Y(t) R_Y(t) + k_{-2} Q_Y^*(t) + k_3 Q_Y^*(t) \\
\frac{d}{dt}M_Y(t) &= k_3 Q_Y^*(t) - k_5 M_Y(t) \\
\frac{d}{dt}P_Y(t) &= k_4 M_Y(t) - k_6 P_Y(t) + (-k_1 D_W(t) P_Y(t) + k_{-1} Q_W(t)) + (-k_1 T_W(t) P_Y(t) + k_{-1} Q'_W(t)) + \\
&\quad (-k_1 D_Z(t) P_Y(t) + k_{-1} Q_Z(t)) + (-k_1 T_Z(t) P_Y(t) + k_{-1} Q'_Z(t))
\end{aligned} \tag{2}$$

$$
\begin{aligned}
\frac{d}{dt}D_Z(t) &= (-k_1D_Z(t)P_Y(t)+k_{-1}Q_Z(t))+(-k_1D_Z(t)P_X(t)+k_{-1}T_Z(t)) \\
\frac{d}{dt}Q_Z(t) &= (k_1D_Z(t)P_Y(t)-k_{-1}Q_Z(t))+(-k_1Q_Z(t)P_X(t)+k_{-1}Q'_Z(t)) \\
\frac{d}{dt}T_Z(t) &= (k_1D_Z(t)P_X(t)-k_{-1}T_Z(t))+(-k_1T_Z(t)P_Y(t)+k_{-1}Q'_Z(t)) \quad (3) \\
\frac{d}{dt}Q'_Z(t) &= (k_1Q_Z(t)P_X(t)-k_{-1}Q'_Z(t))+(k_1T_Z(t)P_Y(t)-k_{-1}Q'_Z(t)) \\
&\quad +(-k_2Q'_Z(t)R_Z(t)+k_{-2}Q^*_Z(t))+(k_9Q^*_Z(t)) \\
\frac{d}{dt}R_Z(t) &= (-k_2Q'_Z(t)R_Z(t)+k_{-2}Q^*_Z(t))+(k_9Q^*_Z(t)) \\
\frac{d}{dt}Q^*_Z(t) &= (k_2Q'_Z(t)R_Z(t)-k_{-2}Q^*_Z(t))+(-k_9Q^*_Z(t)) \\
\frac{d}{dt}M_Z(t) &= k_9Q^*_Z(t)-k_5M_Z(t) \\
\frac{d}{dt}P_Z(t) &= k_4M_Z(t)-k_6P_Z(t)
\end{aligned}
$$

$$
\begin{aligned}
\frac{d}{dt}D_W(t) &= (-k_1D_W(t)P_Y(t)+k_{-1}Q_W(t))+(-k_1D_W(t)P_X(t)+k_{-1}T_W(t)) \\
\frac{d}{dt}Q_W(t) &= (k_1D_W(t)P_Y(t)-k_{-1}Q_W(t))+(-k_1Q_W(t)P_X(t)+k_{-1}Q'_W(t)) \\
\frac{d}{dt}T_W(t) &= (k_1D_W(t)P_X(t)-k_{-1}T_W(t))+(-k_1T_W(t)P_Y(t)+k_{-1}Q'_W(t)) \quad (4) \\
\frac{d}{dt}Q'_W(t) &= (k_1Q_W(t)P_X(t)-k_{-1}Q'_W(t))+(k_1T_W(t)P_Y(t)-k_{-1}Q'_W(t)) \\
&\quad +(-k_2Q'_W(t)R_W(t)+k_{-2}Q^*_W(t))+(k_9Q^*_W(t)) \\
\frac{d}{dt}R_W(t) &= (-k_2Q'_W(t)R_W(t)+k_{-2}Q^*_W(t))+(k_9Q^*_W(t)) \\
\frac{d}{dt}Q^*_W(t) &= (k_2Q'_W(t)R_W(t)-k_{-2}Q^*_W(t))+(-k_9Q^*_W(t)) \\
\frac{d}{dt}M_W(t) &= k_9Q^*_W(t)-k_5M_W(t) \\
\frac{d}{dt}P_W(t) &= k_4M_W(t)-k_6P_W(t)
\end{aligned}
$$
